# Supplementary material for: Content-rich biological network constructed by mining PubMed abstracts
Source: BMC Bioinformatics. 2004 Oct 8;5:147. doi: 10.1186/1471-2105-5-147 (PMC528731; doi:10.1186/1471-2105-5-147)
Supplement: Additional File 2 — The original results of the above study (non-essential files are deleted to keep the file size under the limit set by BMC bioinformatics). [file 1471-2105-5-147-S2.bz2 › chilibotAdditionalFile2/dip40/151ID8479754E546/html/CDC2_CCND1.html]

 


 **CDC2** and **CCND1** 
  
Found 821 abstracts in PubMed, retrieved 40.  
 

 What does Google say? 
 PDF only 
| .edu only 

---

**Interactive relationship** (e.g. stimulation, inhibition, etc)

**Inhibitory relationship**- Cytoplasmic cyclin D  [ **CCND1** ]  1 assembled with cyclin dependent kinase 4 CDK  [ **CDC2** ]  4, and the CDK  [ **CDC2** ]  inhibitors p21Cip1 and p27Kip1.  Ref: 12872751 Kokubyo Gakkai Zasshi, 2003
- Rapamycin and its analogues also prevent cyclin dependent kinase CDK  [ **CDC2** ]  activation, inhibit retinoblastoma protein phosphorylation, and accelerate the turnover of cyclin D1  [ **CCND1** ] , leading to a deficiency of active CDK4 cyclin D1  [ **CCND1** ]  complexes, all of which potentially contribute to the prominent inhibitory effects of rapamycin at the G1 S boundary of the cell cycle.  Ref: 12864941 Clin Breast Cancer, 2003
- Western blotting analysis revealed that the protein levels of cyclin A, cyclin E, and cyclin dependent kinase CDK  [ **CDC2** ]  2 but not cyclin D1  [ **CCND1** ]  and CDK4 decreased after progesterone treatment, but those of CDK  [ **CDC2** ]  inhibitory proteins, p21 and p27, increased.  Ref: 12810531 Endocrinology< MedlineTA>Endocrinology, 2003
- No changes were observed in the expression levels of the G1 regulatory molecules cyclin D1  [ **CCND1** ]  and cdk4, but cell cycle arrest and apoptosis induction was accompanied by accumulation of the cdk  [ **CDC2** ]  inhibitor p21 in both cell lines.  Ref: 12846895 Br J Haematol, 2003
- LY CDK  [ **CDC2** ]  inhibitor treated cells also displayed diminished pRb dephosphorylation on CDK2 and CDK4 specific sites, retinoblastoma protein cleavage, and down regulation of cyclin D  [ **CCND1** ] .  Ref: 12702569 Cancer Res, 2003
**Stimulatory relationship**- The bypass of TNF induced G1 arrest seems to be related to the increase in the stability of cyclin D  [ **CCND1** ]  bound CDK  [ **CDC2** ]  complexes, increasing the total amount of CDK2 4 and 6 complexes and leading to a functional down titration of the p21 waf1 molecules.  Ref: 12761882 J Cell Biochem, 2003
- Transfection of bcl  [ **CCND1** ]  2 in E1A c Ha ras transformants resulted in elevated expression of cyclin cdk  [ **CDC2** ]  complexes inhibitor p21 Waf 1, but not p27 Kip.  Ref: 12722479 Tsitologiia, 2003
**Neutral relationship**

**Non-interactive relationship** (e.g. studied together, co-existance, homology, etc.)

- Overexpression of cyclin D1  [ **CCND1** ]  in these cells makes them insensitive to TNF treatment with the recovery of CDK  [ **CDC2** ]  activity is unable to overcome the inhibitory action of etoposide blocking the cells on G2 M.  Ref: 12761882 J Cell Biochem, 2003
- Co administration of 17 AAG and UCN 01 did not modify expression of Hsp90, Hsp27, phospho JNK, or phospho p38 MAPK, but was associated with further p34  **cdc2**  dephosphorylation and diminshed expression of Bcl  [ **CCND1** ]  2, Mcl 1, and XIAP.  Ref: 12738674 Blood< MedlineTA>Blood, 2003
- Effects of Soy Isoflavones on Apoptosis Induction and G2 M Arrest in Human Hepatoma Cells Involvement of Caspase 3 Activation, Bcl  [ **CCND1** ]  2 and Bcl  [ **CCND1** ]  XL Downregulation, and  **Cdc2**  Kinase Activity.  Ref: 12791511 Nutr Cancer, 2003
- The Gene Ratios c MYC Cyclin dependent Kinase CDK  [ **CDC2** ]  N2A and  **CCND1**  CDKN2A Correlate with Poor Prognosis in Squamous Cell Carcinoma of the Head and Neck.  Ref: 12738730 Clin Cancer Res, 2003
- In a series of 23 surgical biopsies of grade II and III oligodendroglioma, cyclin D1  [ **CCND1** ] , E, A, B1, CDK4 6, CDK2,  **Cdc2**  and p27 Kip.1 have been studied by immunohistochemistry and Western blot.  Ref: 12873740 Neurosci Lett, 2003
- cyclin D1  [ **CCND1** ] , cyclin B, and p34cdc2  [ **CDC2** ]  were examined by RT PCR.  Ref: 12841865 Cancer Sci, 2003
- The linkage of cell cycle and apoptosis has been recognized for c Myc, p53, pRb, Ras, PKA, PKC, Bcl  [ **CCND1** ]  2, NF kappaB, CDK  [ **CDC2** ] , cyclins and CKI.  Ref: 12814432 Cell Prolif, 2003
- Mitogen induced downregulation of the cyclin dependent kinase CDK  [ **CDC2** ]  inhibitor p27 kip1, and induction of the G1 cyclins cyclin D1  [ **CCND1** ] , cyclin A, and cyclin E were also attenuated by the non thiazolidinedione partial PPARgamma agonist.  Ref: 12694805 Eur J Pharmacol, 2003
- Binding of these CKI s to both the cyclin D1  [ **CCND1** ]  CDK  [ **CDC2** ]  4 complex and the cyclin E CDK  [ **CDC2** ]  2 complex were increased and the kinase activities of these complexes were reduced with increasing dietary corticosterone.  Ref: 12807724 Carcinogenesis< MedlineTA>Carcinogenesis, 2003
- Monensin decreased the levels of CDK2, CDK6,  **cdc2** , cyclin A, cyclin B1, cyclin D1  [ **CCND1** ]  and cyclin E proteins but did not alter CDK4 protein.  Ref: 12792794 Int J Oncol, 2003
- Levels of cyclin D1  [ **CCND1** ] , cyclin E, CDK  [ **CDC2** ]  2 and CDK  [ **CDC2** ]  4 were reduced in carcinomas from corticosterone treated rats.  Ref: 12807724 Carcinogenesis< MedlineTA>Carcinogenesis, 2003
- Positive staining for cyclin D1  [ **CCND1** ] , cyclin E, cyclin A, cyclin B1, cdk2, cdk4,  **cdc2** , p53, p21, and p27 was observed in 63%, 66%, 31%, 32%, 51%, 77%, 71%, 43%, 35%, and 60% of the 82 carcinomas, respectively.  Ref: 12792921 Hum Pathol, 2003
- In p18 p21 knockout animals, the G1 phase was shortened as evidenced by an earlier onset of cyclin D  [ **CCND1** ]  and proliferating cell nuclear antigen PCNA expression and cyclin dependent kinase CDK  [ **CDC2** ]  activation after PH.  Ref: 12668976 Hepatology, 2003
- Damaging agents caused p21 Waf 1 and p27 Kip accumulation, but bcl  [ **CCND1** ]  2 overexpression did not restore functions of these inhibitors, since p21 Waf 1 and p27 Kip were unable to suppress cyclin cdk  [ **CDC2** ]  complexes activity after damage.  Ref: 12722479 Tsitologiia, 2003
- These results suggest that the suppression of cell cycle progression in the G1 phase by 5GG was mediated in MCF 7 cells, at least in part, by either the inhibition of cyclin D  [ **CCND1** ]  CDK4 and cyclin E CDK2 activity or the induction of the CDK  [ **CDC2** ]  inhibitors p27 Kip and p21 Cip.  Ref: 12781329 Biochem Pharmacol, 2003
- This study may contribute to the establishment of an animal model for anti cancer research based on cyclin D1  [ **CCND1** ]  suppression or cdk  [ **CDC2** ]  inactivation, or both.  Ref: 12834607 J Comp Pathol, 2003
- The objective of the present study was to analyze the copy number of the frequently amplified oncogenes  **CCND1**  and c MYC in relation to the commonly deleted tumor suppressor gene cyclin dependent kinase CDK  [ **CDC2** ]  N2A p16 to enhance the clinical significance.  Ref: 12738730 Clin Cancer Res, 2003
- The dramatic potentiation of CDK  [ **CDC2** ]  inhibitor induced apoptosis by LY was accompanied by diminished Bad phosphorylation, induction of Bcl  [ **CCND1** ]  2 cleavage, and down regulation of X linked IAP and Mcl 1.  Ref: 12702569 Cancer Res, 2003
